# Poverty screening among paediatricians in the Netherlands: an exploratory study of current practices

**DOI:** 10.1007/s00431-026-07410-7

**Published:** 2026-09-29

**Authors:** Ashley J. P. Smit, Lequisha G. Suisse, Nicole Lucassen, Brita M. de Jong-van Kempen, Koen F. M. Joosten

**Affiliations:** 1 Department of Neonatal and Paediatric Intensive Care, Erasmus Medical Center Sophia Children’s Hospital, University Medical Center Rotterdam, Dr. Molewaterplein 40, Rotterdam, 3015 GD The Netherlands; 2 Department of Patient Care and Quality, Erasmus MC, University Medical Center Rotterdam, Rotterdam, The Netherlands; 3 Erasmus School of Social and Behavioural Sciences, Erasmus University Rotterdam, Rotterdam, The Netherlands; 4 Department of Paediatrics, Frisius Medical Centre Leeuwarden, Leeuwarden, The Netherlands

**Keywords:** Poverty, Child health, Healthcare access, Paediatricians, Screening, Survey

## Abstract

Children growing up in poverty are more likely to experience poorer health. Additionally, families experiencing poverty may be more likely to miss appointments or experience difficulties navigating support services. This highlights a potential role for paediatricians in identifying and addressing poverty, although their practices and perspectives are not well understood. Therefore, this study is aimed at gaining insight into current poverty screening, discussing and referral practices among paediatricians working in the Netherlands. This cross-sectional study distributed a digital survey in 2024 to assess current poverty-related practices among paediatricians in the Netherlands. The Checklist for Reporting Results of Internet E-Surveys was followed in reporting on the study. Descriptive statistics were used to summarise multiple-choice responses. Open-ended responses were analysed using content analysis. Fifty paediatricians completed the survey. Most (92.0%) noticed that patients’ financial concerns negatively affect access to healthcare. Paediatricians reported mixed practices regarding asking patients about financial circumstances and referring to supportive resources, highlighting that it is not part of standard paediatric practice. Various barriers played a role, including a lack of clear referral options, knowledge, identification tools, and conversation tools. Nevertheless, most paediatricians (84.0%) stated that they should play a role in identifying and discussing financial concerns or poverty among their patients.

*Conclusion*: Although paediatricians recognise the importance of poverty for child health and are motivated to address it, they lack the resources for consistent practice. Future research should focus on developing and implementing targeted support strategies to strengthen paediatricians’ capacity to address poverty-related challenges in routine care.

**Supplementary information:**

The online version contains supplementary material available at https://doi.org/10.1007/s00431-026-07410-7.

**Table:** 

**What is Known:** • *Growing up in poverty is associated with poorer health and reduced access to healthcare.* • *Paediatricians can contribute to improving child health outcomes by addressing poverty within clinical care.*
**What is New:** • *Dutch paediatricians recognise the impact of poverty but report inconsistent screening and referral practices.* • *Lack of tools, knowledge, and clear referral pathways limits routine identification and discussion of poverty.*

## Introduction

Growing up in poverty is widely recognised as a key social determinant of health, referring to the conditions in which people are born, grow, work, live and age, and the wider forces that shape the conditions of daily life, and is associated with adverse outcomes across all domains of child development, including physical health, cognitive development, socio-emotional functioning, behavioural outcomes, and mental wellbeing [1–4]. In the Netherlands, approximately 10% of children grow up in households experiencing poverty or almost poverty [5]. While this figure reflects income-based definitions of poverty, poverty extends beyond income alone; material deprivation and subjective financial strain can occur independently of income level and shape parental and child wellbeing in similar ways [6–9]. Therefore, in this paper, the term poverty is used as an umbrella concept encompassing not only low household income but also material deprivation and subjective financial strain, which may occur separately or in combination, adopted for clarity and consistency throughout the paper. Children growing up in poverty, in this broader sense, are more likely to report poorer health, live with chronic conditions, and make greater use of healthcare services than children growing up in financially secure households [10].

Besides its association with child development, poverty is also associated with inequalities in access to and use of social care, preventative care, and healthcare services [11–13]. Even with a system of universal health insurance, unequal opportunities for families are embedded in the way the healthcare and insurance system are organised. As a result, families experiencing poverty may be more likely to delay seeking care, miss appointments, face transportation or logistical barriers, or experience difficulties navigating support services [12, 14].

The relationship between poverty and child development is complex and multifactorial [15]. Poverty itself is largely influenced by structural and systemic factors, such as labour market conditions, social security systems, and discrimination, which lie largely outside the control of individual families. The detrimental effects of growing up in poverty can persist into adulthood, affecting health, behaviour, and socioeconomic outcomes and contribute to the intergenerational persistence of poverty [16–19].

While addressing child poverty requires structural and policy-level solutions, paediatric healthcare professionals occupy a position that may allow them to recognise signs of poverty and offer support at the individual level. Given the close relationship between poverty and child health, literature and policy statements suggest that paediatricians can contribute to improving child health outcomes by identifying poverty-related stressors, initiating non-stigmatising conversations, and connecting families to appropriate resources [20–22]. This role aligns with the principles of the United Nations Convention on the Rights of the Child, which recognises children’s right to the highest attainable standard of health and access to healthcare services [23]. Previous research, mostly US-based, has explored paediatricians’ perspectives on poverty-related practices (e.g. screening, discussing and referring) [24–26]. These studies suggested that paediatricians generally support screening for and addressing poverty but that there are various barriers and concerns hindering a structural approach, including the feasibility of such practices and the responsibility of paediatricians. However, little is known about how paediatricians in the Netherlands currently perceive poverty among their patients or which screening, discussion, and referral practices they engage in in daily clinical care.

### The current study

To gain insight into current poverty screening, discussing and referral practices among paediatricians, we conducted a nationwide digital survey among paediatricians with a focus on those working within hospitals in the Netherlands.

## Methods

### Study design

This cross-sectional study distributed a digital survey to assess current practices related to poverty screening, discussion, and referral among paediatricians in the Netherlands. The Checklist for Reporting Results of Internet E-Surveys was followed in reporting on the study [27].

### Participants and recruitment

Participants were a convenience sample of paediatricians and paediatric residents working within hospitals in the Netherlands. To ensure survey response by the targeted audience, the first two survey questions asked respondents to indicate their affiliated hospital and position (Supplementary file 1). There were no inclusion or exclusion criteria other than being a paediatrician or paediatric resident.

A link to the digital survey was distributed via the website and e-mailed newsletter of the Dutch Association of Paediatrics (Nederlandse Vereniging van Kindergeneeskunde; NVK). These contained background information on the relation between poverty and child health and the aim of the study (Supplementary file 2). The newsletter was sent to all members of the NVK. In addition, a QR code linking to the survey was distributed during the 2024 NVK Annual Conference. The survey was open from June to November 2024. Participation was voluntary and anonymous, and no incentives were offered. The survey was initiated 77 times and submitted 50 times, resulting in a completion rate of 64.9%.

### Survey

The survey (Supplementary file 1) was developed to gain insight into current poverty screening, discussing and referral practices among paediatricians, with a particular focus on hospital-based paediatric care. It began with the following definition of poverty: *poverty exists when material, cultural, and social resources are insufficient to live in a way that is considered minimally acceptable in the society in which one lives*. It consisted of 11 items: 2 items on professional background; 8 items on poverty screening, discussing, and referral; and an open question inviting respondents to share anything related to the topic of poverty practices. The survey was self-developed by a team of researchers and paediatricians and tailored to the context of paediatrics. It was piloted with five other paediatricians, whose feedback was used to refine the final version. They were not part of the final sample. Most questions were mandatory, as indicated in Supplementary file 1. Some questions were conditionally displayed, depending on previous responses. The questions included multiple-choice and open-ended formats. The final question was an open-ended question, inviting respondents to share any additional comment. No randomisation of question order or response options was used. Respondents could not review or change their answers after submitting the survey. The mean completion time was 5 min and 19 s.

The survey was administered using Limesurvey. The survey was open access; no registration or login was required to access the survey. There were no measures that prevented multiple submissions from the same device.

### Ethical considerations

Ethical review was not sought because the study involved an anonymous, non-invasive survey. No IP addresses were collected, and the results were securely stored on an internal drive. Respondents were informed about the purpose of the study, that participation was voluntary and that the findings would be disseminated.

### Data analysis

Data were exported to and analysed using Excel. Only submitted surveys (*n* = 50) were included in the analysis. Incomplete entries consisted of fully empty records (*n* = 27) and were excluded. Descriptive statistics were used to summarise multiple-choice responses. Open-ended responses were analysed using content analysis [28]. Responses were reviewed and coded by AJPS into categories based on similarity in content, with categories developed inductively from the data. The coding scheme was iteratively refined during the analysis process. To enhance reliability of the analysis, a second researcher, LGS, reviewed the coding framework and coded responses, and discrepancies were resolved through discussion until consensus was reached. Category frequencies were calculated to descriptively summarise the distribution of responses.

## Results

### General

The survey was completed by 50 paediatricians who were working in 25 different hospitals and one public health organisation in the Netherlands. Their roles were as follows: general paediatricians (*n*=21, 42.0%), academic paediatricians (*n*=13, 26.0%), residents (*n*=9, 18.0%) and sub-specialists (*n*=7, 14.0%). Of the respondents, 24 (48.0%) worked in the three largest Dutch cities (each with more than 500,000 inhabitants), 14 (28.0%) in large cities (100,000–250,000 inhabitants), and 12 (24.0%) in medium or small cities (fewer than 100,000 inhabitants).

### Poverty indicators

**Fig. 1:**
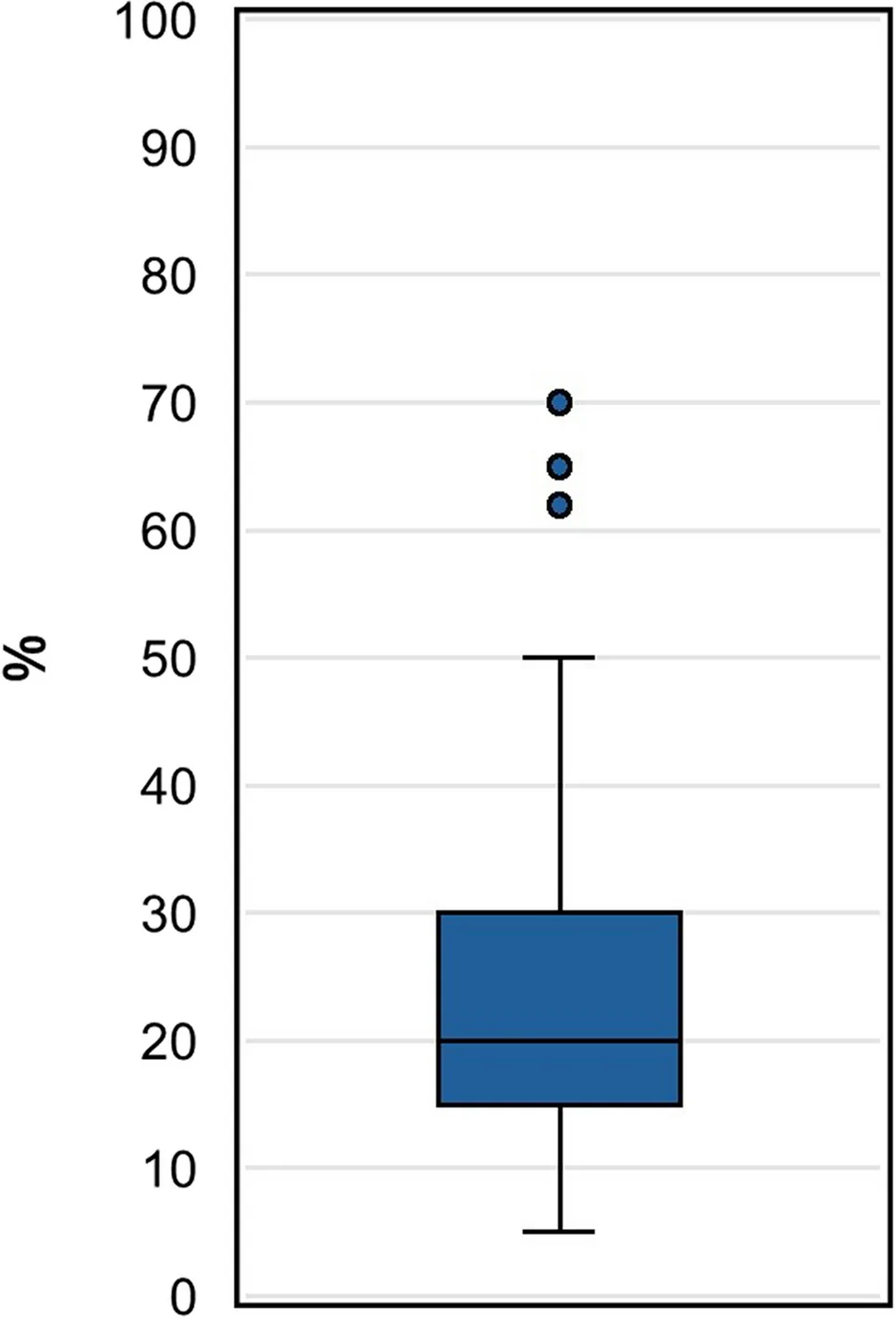
Paediatricians’ estimates of percentage of patients growing up in poverty (*N* = 50)

The respondents suspect that 5.0 to 70.0% of their patients grow up in a family with financial concerns or poverty, with a median of 20.0% (see Fig. 1). Most respondents (*n* = 46, 92.0%) noticed or suspected that poverty negatively affects access to preventive services and healthcare, with one respondent stating: “*Do more research on how poverty stands in the way of access to care! [It] is a bigger problem than people think…*”.

**Fig. 2:**
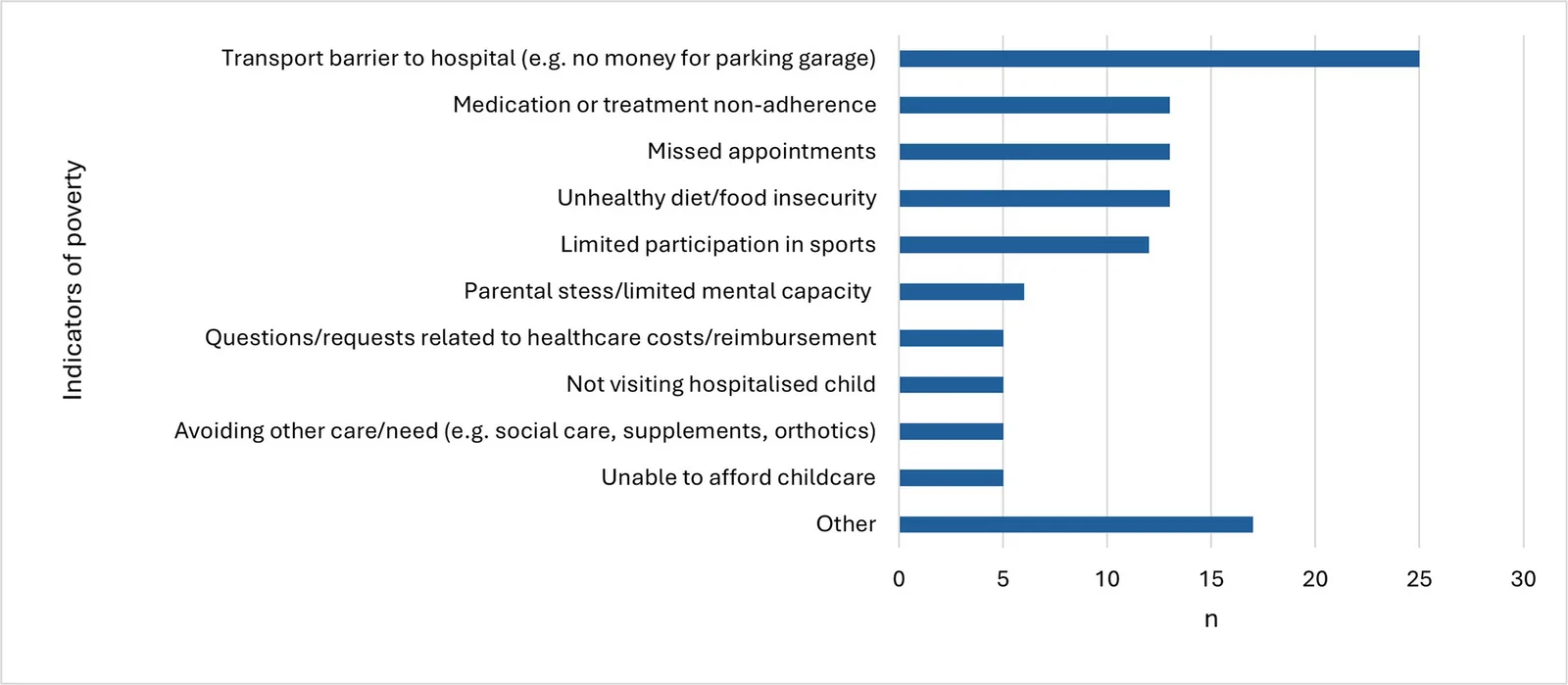
Number of paediatricians reporting each indicator of poverty (*N* = 43)

Of these 46 respondents, 43 (93.5%) provided various signs or situations indicative of poverty. Of these respondents 39 (90.7%) provided more than one example. Their responses were categorised (see Fig. 2). The most reported indicator was constraints in transportation to the hospital (*n* = 25, 58.1%). These constraints included not owning a car, not being able to afford public transportation or gas, and not being able to afford parking costs. Transportation constraints, but also the inability to afford childcare and parental stress, sometimes resulted in missed or cancelled appointments (*n* = 13, 30.2%), or parents not being able to visit their hospitalised child (*n* = 5, 11.6%). As one respondent described: “*On the day of the operation, the parents could not be present because the travel costs were too high for them. Heartbreaking*”. Other frequently mentioned indicators related to children’s lifestyle (diet, *n* = 13, 30.2%; physical activity, *n* = 12, 27.9%). Additionally, medication or treatment non-adherence was a frequently noted indicator (*n* = 13, 30.2%), often due to associated costs. Notably, respondents shared that in some cases parents explicitly shared with them that costs were a concern (e.g. “I can’t afford transportation”), whereas in other cases, situations such as not showing up for an appointment and not collecting prescribed medication implicitly suggested financial concerns. Seventeen respondents shared indicators that were grouped under “other” in Fig. 2, as they were each reported by four or fewer respondents. These are presented in Supplementary file 3.

### Paediatrician’s poverty screening and professional role

Twenty-one respondents (42.0%) reported that they sometimes ask the parents of their patients about their financial situation. Ten (20.0%) indicated that they frequently ask about this, and two (4.0%) stated that they always do so. Eleven respondents (22.0%) rarely inquire about financial concerns, and six (12.0%) reported that they never ask.

Regarding the role of a paediatrician in addressing poverty amongst their patients, 42 respondents (84.0%) stated that paediatricians should play a role in identifying and discussing financial concerns or poverty among their patients, consider (affordable) solutions, or refer families to available support services. Some of them indicated that they perceive a role primarily when financial problems have a direct impact on the child’s health or development, access to care or treatment. The remaining eight paediatricians (16.0%) were uncertain about their role in addressing financial difficulties. Their uncertainty was based on experiences in which parents denied financial problems, as well as the belief that they themselves were unable to offer effective solutions, or due to workload constraints. As one paediatrician noted: “*It is quite difficult to determine where the responsibility lies, as we already have a signalling role for many issues as paediatricians (e.g. vaccination, lifestyle, *etc*.)*”. Some expressed that the responsibility should instead or preferably lie with schools, public youth healthcare, childcare organisations, medical social workers, or psychosocial care providers. Three respondents additionally shared that poverty is a socio-political matter, and paediatricians should participate in the wider societal debate surrounding poverty and healthcare.

**Fig. 3:**
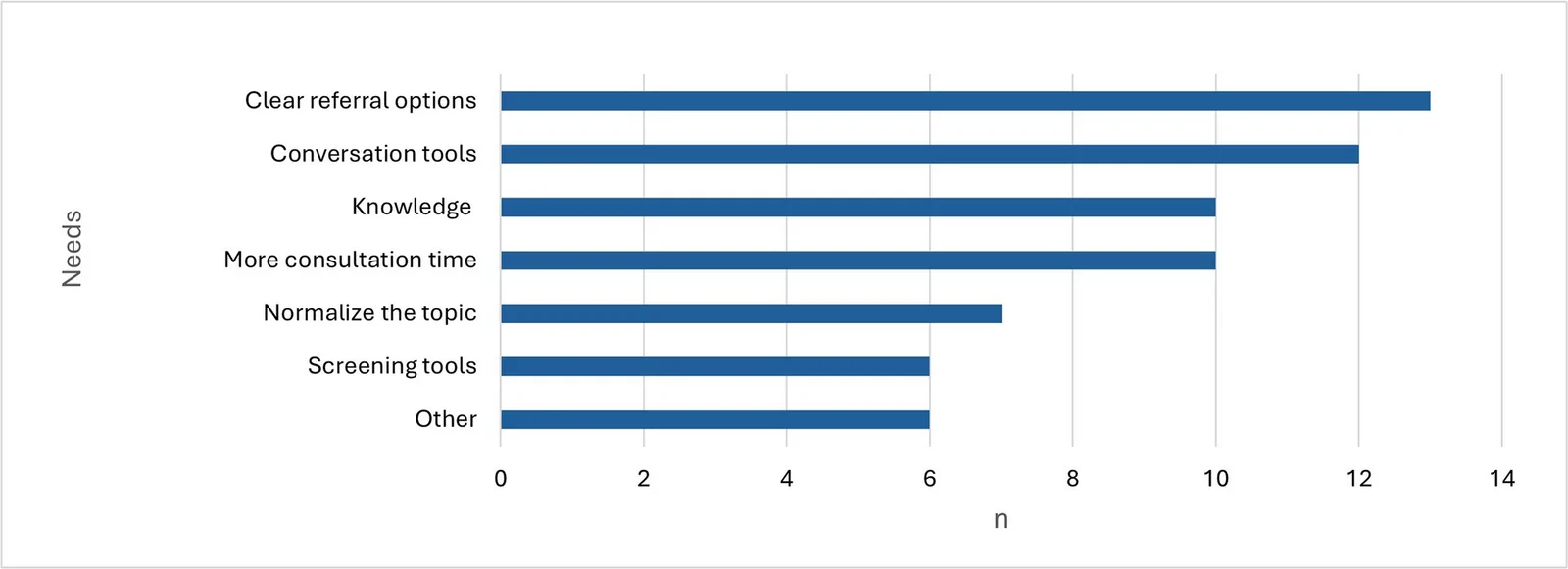
Number of paediatricians reporting each need to facilitate poverty practices (*N* = 43)

Paediatricians indicated that, to facilitate poverty screening and discussion, they would benefit from clear and appropriate referral options (*n* = 13, 30.2%; see Fig. 3), as they wish to be able to offer adequate support to these patients. One respondent noted: “*The only question is whether we have a solution [for poverty related problems]; then I would need to know whom I can refer these people to for this*”. Further normalisation of the topic was also perceived beneficial (*n* = 6, 14.0%). In addition, respondents expressed a need for tools to facilitate the initiation of conversations about financial difficulties with patients or their parents (*n* = 12, 27.9%). They also reported requiring more time during consultations to address this topic (*n* = 10, 23.3%), more knowledge (*n* = 10, 23.3%), including knowledge regarding the steps after identification of poverty (*n* = 5, 11.6%) and on the signs of poverty (*n* = 2, 4.7%), and screening tools such as a questionnaire (*n* = 6, 14.0%). Six respondents (14.0%) had needs that were grouped under “other”, as they were mentioned by two or fewer respondents. These needs are presented in Supplementary file 3. Four paediatricians answered that screening and discussing could be addressed by simply asking about poverty, of which two did not report any need as described above. Five paediatricians did not answer this question or reported that they did not know how to answer.

### Referring patients in poverty

Forty percent of paediatricians (*n* = 20) reported referring their patients when there is a suspicion of financial difficulties or poverty. However, nearly half (*n* = 24, 48.0%) indicated that their decision to refer depends on contextual factors, such as the availability of referral options and the presence of existing support within the family. Some paediatricians reported referring only when patients or their parents are open to discussing financial matters, or when financial difficulties are perceived to impede the patient’s treatment or development, with one respondent stating: “*It depends on the severity, as well as the relationship with the child’s medical condition*”*.*

Paediatricians reported referring to a range of resources, including medical social work, the general practitioner (GP), public youth healthcare, and local multidisciplinary community support teams. In addition, some mentioned local organisations and national funds that support participation in sports, birthdays or access to food. Nevertheless, medical social work seems the most straightforward referral option, as highlighted by one respondent: “*We consult medical social work on the ward, but I’m not very familiar with the other options*”.

A minority of paediatricians (*n* = 6, 12.0%) indicated that they do not refer patients when financial difficulties or poverty are suspected. Most of them (*n* = 5, 83.3%) reported lacking knowledge about referral options or how to provide appropriate support, while one paediatrician considered this outside the scope of a paediatrician's responsibilities. In these cases, the GP or a community service desk were mentioned as more appropriate sources of support.

### Perspectives, experiences, and observations

Paediatricians were invited to share their thoughts in an open-ended question. Paediatricians shared a range of perspectives. First, eleven respondents (22.0%) praised the attention given to research on poverty in paediatric care describing it as an urgent and relevant issue. Second, paediatricians emphasised the need for greater awareness of the impact of poverty on children’s development and access to healthcare (*n* = 5, 10.0%), as well as more training and practical tools to support screening and conversations about poverty (*n* = 6, 12.0%), with one stating: “*Guidance to help make this discussable is very welcome*”. Some paediatricians reflected on their own screening practices or shared examples of families experiencing poverty (*n* = 5, 10.0%). In addition, the importance of or greater attention to low (health) literacy was also mentioned as a related challenge in communication and care provision (*n* = 1, 2.0%). Finally, one paediatrician (2.0%) noted that poverty may shape healthcare-seeking or accepting behaviour in less expected ways, for example when families request hospital admission for access to free meals.

## Discussion

### General findings

This study examined the self-reported practices related to poverty screening, discussion, and referral among 50 paediatricians and paediatric residents in the Netherlands. Our survey explored several domains, including the suspected prevalence of poverty in paediatric healthcare, the perceived impact of poverty on child health and healthcare access, barriers and facilitators to poverty-related practices, views on professional responsibility, and the normalisation of poverty-related conversations. While addressing child poverty ultimately requires structural and policy-level interventions, the findings of this study highlight the potential role of paediatric healthcare professionals at the individual level. Their close contact with families places them in a unique position to recognise poverty-related stressors, engage in conversations, and facilitate access to appropriate resources. In line with previous international research, our results suggest general support among Dutch paediatricians for such practices [24–26]. However, multiple barriers limit the structural identification of poverty in patients and the initiation of discussions with families.

### Suspected poverty prevalence in paediatric healthcare

Respondents estimated that, on average, 20% of their patients and families experienced poverty or financial difficulties. However, the reported prevalence varied widely between respondents (5 to 70%). This variation may partly reflect differences in hospital catchment areas, as poverty rates differ considerably across Dutch municipalities and tend to be higher in large urban areas [5]. However, substantial variability was also observed within paediatricians working in the largest cities [data not shown], suggesting that geographical location alone does not fully explain the differences. Other factors, such as the disease profile of the patient population and healthcare provider’s professional background and exposure to poverty-related training, may influence perceived poverty prevalence as well [29–31]. Nevertheless, these results show that paediatricians encounter children living in poverty, either occasionally or on a regular basis, and identify it to a greater or lesser extent.

### Impact of poverty on paediatric healthcare

Almost all respondents noticed or suspected that the financial concerns patients and their parents deal with result in decreased access to prevention and healthcare**.** In addition, respondents provided numerous examples that highlight the impact poverty has on both child health and access to healthcare, in line with the literature [1–3, 11–13]. Though the Dutch healthcare insurance system covers most healthcare services for children, our results suggest that financial concerns and material hardship may continue to hinder families from following medical advice, including the use of non-reimbursed medications or supplements and the adherence to lifestyle advice. In addition, insurance rarely covers healthcare-related transportation costs, which was the most frequent given example by respondents of how poverty restricts access to healthcare. Transportation costs as a barrier to attend medical appointments have been previously identified among paediatric healthcare providers as an important obstacle to care [32].

In addition, respondents also noted that parents of paediatric patients who experience poverty often have limited psychological bandwidth due to ongoing financial concerns, along with the stress it causes. As a result, missed appointments and difficulties adhering to care plans were observed. These patterns align with the literature, which shows that poverty-related stress reduces executive functioning, disrupts daily routines, and increases the likelihood of non-attendance in healthcare [33]. Poverty may also affect child health through chronic stress and increased exposure to adverse childhood experiences. These broader psychosocial pathways were beyond the scope of the current study, but their impact warrants consideration in clinical practice.

### Barriers and facilitators to poverty practices

Respondents reported several barriers to poverty-related practices, including insufficient knowledge and resources and a lack of time, consistent with the literature [34]. Our results showed that two-thirds of respondents ask about the financial concerns of their patient’s family, though not necessarily structurally. In addition, we showed that many paediatricians refer families with financial concerns for support. Among those who did not, this was primarily attributed to a lack of knowledge about available resources and referral pathways, rather than reluctance to provide such support.

Training emerged as an important facilitator, as many respondents indicated a need for greater knowledge to support poverty screening, discussion, and referral. In addition, respondents expressed a need for practical screening and conversation tools. Studies show that training and screening- and conversation tools improve discussing, identifying and referral of poverty in patients among healthcare providers [35]. Any screening tool implemented in paediatric practice should not only support the identification of financial concerns, but also be feasible within routine clinical workflows and acceptable to both families and healthcare professionals [35]. Furthermore, paediatricians may benefit from an integrated referral system, with clear referral structures and multidisciplinary collaboration, to help translate identification of poverty into meaningful support for families [36, 37]. Therefore, investing in targeted training, the development, and implementation of accessible screening and conversation tools, particularly those adapted to paediatric practice and local referral systems, and implementation of an integrated referral system, is recommended to facilitate poverty practices in paediatric healthcare providers.

### Professional responsibility and shared care

Beyond individual barriers, our findings highlight an important question regarding professional responsibility. While previous literature often assumes that paediatricians should address poverty as part of their provided care [20, 21, 38], it is not entirely clear whether Dutch paediatricians agree with this. Our results show that most respondents think they should play a role in identifying and discussing financial concerns or poverty among their patients, considering (affordable) solutions, and referring families to appropriate resources.

Nevertheless, some paediatricians questioned whether addressing financial concerns falls within the scope of their responsibilities and instead suggested this role might be better suited to social workers or GPs. However, given the well-established impact of poverty on child health and development, poverty constitutes a key social determinant of health and therefore a legitimate paediatric concern. Although general practitioners function as central gatekeepers in the Dutch healthcare system, this does not negate paediatricians’ responsibility. In some cases, poverty may only become visible at the paediatric care level, particularly when care becomes more complex or prolonged. Rather than specifying the role to a single professional group, poverty practices should be a shared-care approach, with professionals across disciplines working together. While this study focused on paediatricians, future research should broaden its scope to include other healthcare professionals involved in child healthcare, such as the general practitioner, nurses, emergency medicine physicians, medical social workers, dentists and youth healthcare professionals. Such research should not only examine current practices but also explore how different healthcare professionals can collaborate in identifying and addressing poverty among families. At the same time, it is important to acknowledge that identifying financial concerns and referring families to supportive resources, while valuable, do not constitute a structural solution to poverty. Screening and referral should be seen as entry points rather than endpoints. Future research should therefore examine what happens after referral, including whether families are able to access the suggested resources, whether the support aligns with their needs, and whether it leads to meaningful improvements in their circumstances. Evaluating the effectiveness and perceived usefulness of follow-up support could help strengthen poverty-related practices and ensure they translate into tangible benefits for families. In addition, research exploring the experiences and perspectives of patients and their families regarding poverty-related practices in paediatric healthcare is needed to better understand the acceptability of these practices.

### Normalisation of poverty-related conversations

Although many respondents indicated a need for specific conversation tools to support discussions about financial concerns or poverty, a subset of respondents reported that they did not require additional resources but needed to proactively initiate the conversation (“just ask”). This suggests that barriers to addressing poverty in clinical practice may not be solely related to a lack of knowledge or tools but may also reflect discomfort on the part of healthcare professionals. This interpretation is further supported by respondents’ emphasis on the need to normalise discussions about poverty as a prerequisite for effective screening and referral. Qualitative studies show that most parents are comfortable discussing poverty when these conversations are embedded in routine care, clearly linked to the child’s health, and approached in a non-judgemental and empathetic manner [39–41]. Therefore, embedding such conversations into routine care may help reduce provider discomfort and reposition the topic as a standard and expected component of paediatric practice.

### Strengths and limitations

This study has several strengths. First, the survey sample consisted of paediatric residents, academic paediatricians, sub-specialists, and general paediatricians from all regions of the Netherlands, providing a diverse range of perspectives. Second, the survey addressed multiple aspects of the role of healthcare professionals in addressing poverty, including screening, discussing poverty-related issues, and referral practices. This comprehensive scope provides a nuanced insight into Dutch paediatricians’ perspectives on poverty in healthcare. Third, the inclusion of open-ended questions allowed respondents to elaborate on their answers, thereby adding qualitative depth to the findings.

However, some limitations should be acknowledged. First, this study used a self-developed survey. Although pilot-tested for clarity, relevance, and feasibility, it was not formally validated. As such, the survey is not suitable as a validated measure of intervention or policy effects and findings should be interpreted as exploratory only. Future research using this instrument for evaluative purposes should include formal psychometric validation. Second, the survey relied on self-reported data regarding paediatric healthcare providers’ poverty-related practices, which may not fully reflect actual behaviour in healthcare setting. Third, although our sample was diverse, the total number of respondents was limited. National data indicate that over 1800 paediatricians are registered in the Netherlands [42], suggesting that our sample represents only a fraction of the target population. In addition, as with many survey-based studies, there is a potential risk of response bias. Paediatricians with a greater interest in or awareness of poverty-related issues may have been more likely to participate. This could have resulted in an overestimation of awareness, engagement in poverty related practices and positive attitudes toward addressing poverty in paediatric healthcare providers.

## Conclusion

This exploratory study suggests that poverty is a salient issue within paediatric healthcare that affects healthcare access, treatment adherence, and child health. Responding paediatricians recognise the relevance of family financial circumstances for child health and express a strong motivation to play a more active role in addressing these concerns in the clinical setting. However, despite this willingness, respondents report insufficient practical tools, training, and clear referral pathways to effectively identify, discuss, and address poverty-related challenges. These findings highlight opportunities to better support paediatricians translate their motivation into consistent and structured practice. Future research should therefore focus on implementing and evaluating targeted support for paediatric healthcare providers in poverty-related practices, as well as assessing the effectiveness and perceived usefulness of follow-up support.

## Supplementary information

Below is the link to the electronic supplementary material.

Supplementary file1 (DOCX 33 kb)

Supplementary file2 (XLSX 25.9 KB)

## Data Availability

Aggregated results are presented in the manuscript and supplementary materials. Individual survey responses are not publicly available due to privacy considerations.

## Abbreviations

GP: General practitioner
NVK: Dutch Society for Paediatrics

## Declarations

### Disclaimer

During the preparation of this work the authors used Microsoft Copilot GPT-5 Chatmodel to improve readability of the manuscript. After using this tool, the authors reviewed and edited the content as needed and take full responsibility for the content of the published article.
