# Supplementary material for: Poverty screening among paediatricians in the Netherlands: an exploratory study of current practices
Source: Eur J Pediatr. 2026 Sep 29;185(10):784. doi: 10.1007/s00431-026-07410-7 (PMC13624033; doi:10.1007/s00431-026-07410-7)
Supplement: Supplementary file 1 [file 431_2026_7410_MOESM1_ESM.docx]

**Supplementary information**

**Supplementary file 1 – The survey**
**Supplementary file 2 – The invitation to the survey**
**Supplementary file 3 – Answers to the survey including answers categorised as ‘other’**

**Appendix 1 – The survey**

**Poverty Screening**
Poverty is present when material, cultural, and social resources are insufficient to live in a way that is considered minimally acceptable in the society in which one lives.

There are 11 questions in this survey.

1. **Which hospital do you work in?***
   *Open-ended question*
2. **What is your (sub)specialty?***

- General paediatrician
- Academic paediatrician
- Resident
- Subspecialist in a general hospital
- Other

3. **In what percentage (%) of your patients do you suspect they are dealing with financial concerns or poverty?***
   *Open-ended question*
4. **Do you notice or suspect that financial concerns or poverty among your patients leads to poorer access to prevention and care?***

- Yes, I notice or suspect this
- No, I do not notice or suspect this
- I do not know

*Question 5 is only shown if question 4 was answered with “Yes, I notice or suspect this”.*

5. **Can you give one or more examples of a situation in which you noticed or suspected this?**
   *Open-ended question*
6. **Do you ask about the financial situation or financial concerns of (the parents of) your patients?***

- I always ask about this
- I regularly ask about this
- I sometimes ask about this
- I almost never ask about this
- I do not ask about this
- Other

7. **What do you consider to be the role of the paediatrician in discussing financial concerns or poverty with their patients?***
   *Open-ended question*
8. **What would you need as a paediatrician to identify financial concerns or poverty among your patients?***
   *Open-ended question*
9. **Do you refer your patients, or would you refer them, in cases of (suspected) financial concerns or poverty?***

- Yes, I refer
- No, I do not refer
- It depends/varies

*Question 10a is only shown if question 9 was answered with “Yes, I refer”.*

- **10a. To whom or to what do you refer / would you refer in cases of (suspected) financial**
  **concerns or poverty?***
  *Open-ended question*

*Question 10b is only shown if question 9 was answered with “No, I do not refer”.*

- **10b.** **Why do you not refer / would you not refer in cases of (suspected) financial concerns or**
  **poverty?***
  *Open-ended question*

*Question 10c is only shown if question 9 was answered with “It depends/varies”.*

- **10c. Can you explain your previous answer? When do you refer and when not in cases of**
  **(suspected) financial concerns or poverty?***
  *Open-ended question*
- **11. Would you like to share anything else on this topic?**
  *Open-ended question*

* Indicates a mandatory question

**Appendix 2 – The invitation to the survey**

In the Netherlands, an increasing number of children are growing up in poverty or with financial worries. Growing up in poverty and the associated chronic stress within the family can have consequences for children's lifestyles, as well as their mental and physical health. Conversely, a child's illness can lead to financial worries, for example, because parents have less time to work.

**Short online questionnaire**
To investigate this, colleague Prof. Dr. Koen Joosten (paediatric intensivist Neonatal & Paediatric Intensive Care, Erasmus MC), together with the research group of Prof. Nicole Lucassen (Professor of Child Poverty at Erasmus University Rotterdam) and the NVK Advocacy Committee, has developed a short online questionnaire. They would greatly appreciate it if you would take the time to complete this short questionnaire.

**Curious about your experiences in daily practice**
They would like to hear about your experiences as a paediatrician regarding poverty in relation to your patients' health. Do you ever suspect poverty, or do you notice it? What were the signs you noticed, and how did you subsequently deal with them? Additionally, we are interested in your opinion regarding the role of paediatricians in identifying poverty among patients. Do you believe that paediatricians should play an active role in this?

On behalf of the research group, thank you very much for your cooperation! We will provide feedback on the results at a later date.

*The original Dutch invitation can be found through the following link:*
[*Armoede signaleren: survey - De Nederlandse Vereniging voor Kindergeneeskunde*](https://www.nvk.nl/nieuws/armoede-signaleren-survey/)

**Supplementary file 3 – Answers to the survey including answers categorised as ‘other’**

**Indicators of financial concerns as answered to question 5**

| **Indicator** | | **Number of paediatricians reporting the indicator** |
| --- | --- | --- |
| Healthcare access and use | Missed appointments | 13 |
|  | Medication or treatment non‑adherence | 13 |
|  | Transport barrier to hospital (e.g. no money for parking garage) | 25 |
|  | Avoiding other care/need (e.g. social care, supplements, orthotics) | 5 |
|  | Not visiting hospitalised child | 5 |
|  | Questions/requests related to healthcare costs/reimbursement | 5 |
| Material, resource and participation deprivation | Unhealthy diet/food insecurity | 13 |
|  | Poor housing conditions* | 2 |
|  | Inability to secure appropriate housing* | 1 |
|  | Unable to afford childcare | 5 |
|  | No phone credit to call hospital* | 1 |
|  | Child does not own a bike* | 2 |
|  | Limited participation in sports | 12 |
|  | Child does not bring lunch to school* | 1 |
| Explicit indicators of financial vulnerability | Explicit statement from parents about not having money* | 1 |
|  | Food bank use* | 1 |
|  | Being under financial administration* | 1 |
|  | Victim of benefits scandal* | 1 |
|  | Fear of job loss* | 1 |
| Observable characteristics | Poor hygiene* | 1 |
|  | Under/overweight child* | 3 |
|  | Parental stress/limited mental capacity | 6 |
|  | Smoking (and unable to quit)* | 3 |
|  | Inadequate or broken clothing or items* | 3 |

* Indicators grouped under ‘Other’ in Figure 2.

**Needs of paediatricians as answered to question 8**

| **Need** | **Number of paediatricians reporting the need** |
| --- | --- |
| Clear referral options | 13 |
| Conversation tools | 12 |
| Knowledge | 10 |
| More consultation time | 10 |
| Normalize the topic | 7 |
| Screening tools | 6 |
| E-learning* | 2 |
| A team that includes social workers* | 2 |
| Trust it will be appreciated* | 1 |
| Delegate to colleague* | 1 |
| Getting a better feel for the signs of poverty* | 1 |
| Awareness * | 1 |
| Prioritise* | 1 |

* Indicators grouped under ‘Other’ in Figure 3.
